# Evaluation of A-Site Ba^2+^-Deficient Ba_1−*x*_Co_0.4_Fe_0.4_Zr_0.1_Y_0.1_O_3−*δ*_ Oxides as Electrocatalysts for Efficient Hydrogen Evolution Reaction

**DOI:** 10.1155/2018/1341608

**Published:** 2018-09-12

**Authors:** Xiangnan Li, Liqing He, Xiongwei Zhong, Jie Zhang, Shijing Luo, Wendi Yi, Luozheng Zhang, Manman Hu, Jun Tang, Xianyong Zhou, Xingzhong Zhao, Baomin Xu

**Affiliations:** ^1^Department of Materials Science and Engineering, Southern University of Science and Technology, Shenzhen, Guangdong 518055, China; ^2^Department of Physics, Wuhan University, Wuhan, Hubei 430072, China

## Abstract

Exploring earth-abundant and cost-effective catalysts with high activity and stability for a hydrogen evolution reaction (HER) is of great importance to practical applications of alkaline water electrolysis. Here, we report on A-site Ba^2+^-deficiency doping as an effective strategy to enhance the electrochemical activity of BaCo_0.4_Fe_0.4_Zr_0.1_Y_0.1_O_3−*δ*_ for HER, which is related to the formation of oxygen vacancies around active Co/Fe ions. By comparison with the benchmarking Ba_0.5_Sr_0.5_Co_0.8_Fe_0.2_O_3−*δ*_, one of the most spotlighted perovskite oxides, the Ba_0.95_Co_0.4_Fe_0.4_Zr_0.1_Y_0.1_O_3−*δ*_ oxide has lower overpotential and smaller Tafel slope. Furthermore, the Ba_0.95_Co_0.4_Fe_0.4_Zr_0.1_Y_0.1_O_3−*δ*_ catalyst is ultrastable in an alkaline solution. The enhanced HER performance originated from the increased active atoms adjacent to oxygen vacancies on the surface of the Ba_0.95_Co_0.4_Fe_0.4_Zr_0.1_Y_0.1_O_3−*δ*_ catalyst induced by Ba^2+^-deficiency doping. The low-coordinated active atoms and adjacent oxygen ions may play the role of heterojunctions that synergistically facilitate the Volmer process and thus render stimulated HER catalytic activity. The preliminary results suggest that Ba^2+^-deficiency doping is a feasible method to tailor the physical and electrochemical properties of perovskite, and that Ba_0.95_Co_0.4_Fe_0.4_Zr_0.1_Y_0.1_O_3−*δ*_ is a potential catalyst for HER.

## 1. Introduction

The hydrogen fuel cell is considered as one of the most promising green solutions for new energy vehicles with the advantages of high working efficiency and zero emission [1–3]. Electrochemical water splitting is an efficient and promising energy storage technology to produce pure H_2_, benefiting from abundant water resources on the earth, via converting electrical energy generated from intermittent wind energy and solar energy into chemical energy [4, 5]. In the practical application of alkaline water electrolysis, it is still a great challenge to develop a highly efficient catalyst with low cost and good electrochemical stability for H_2_ production. Though carbon-supported Pt (Pt/C) catalysts are reported to have the highest activity toward hydrogen evolution reaction (HER), their widespread application is limited by their high cost, low crust abundance, and poor stability [6]. Therefore, the development of cost-effective and earth-abundant catalyst materials for HER with high activity and stability is of significant importance for realizing large-scale pure hydrogen production through alkaline water electrolysis.

Very recently, several nonnoble functional heterojunction-like-structured electrocatalysts, including metal/metal oxide/carbon hybrids [7, 8], transition-metal sulfides [9], and nitrides [5], have been reported to exhibit outstanding catalytic activities for HER. On these heterojunction-like-structured interfaces, positively-charged metal ion species could preferentially serve as an adsorption site for OH^−^ (generated by H_2_O splitting) due to the strong electrostatic affinity between each other, while a nearby metal or anion ion site would be kinetically beneficial for the adsorption of H. Consequently, these heterojunctions of metal cation/metal atom or anion are able to function synergistically in order to facilitate the Volmer process and thus render stimulated HER catalytic activity [7]. Benefiting from the advantage of flexibility in the oxidation states of transition metals and high tolerance of defective structures for oxygen vacancy or excess, the perovskite oxides with a general formula ABO_3−*δ*_ (where A = rare earth or alkaline earth metal ions and B = transition-metal ions) can be engineered to fit a wide range of applications [10–12]. In a perovskite structure, the octahedral building containing a transition-metal cation and contiguous 6-fold coordinated oxygen anions could play the role of heterojunctions and possibly be reactive sites for HER. Very recently, the perovskite oxides Ba_0.5_Sr_0.5_Co_0.8_Fe_0.2_O_3−*δ*_, Pr_0.5_(Ba_0.5_Sr_0.5_)_0.5_Co_0.8_Fe_0.2_O_3−*δ*_, and SrNb_0.1_Co_0.7_Fe_0.2_O_3−*δ*_ were found to be highly active and stable for HER [13, 14], which demonstrate the remarkable effectiveness of perovskite oxides as candidates for the HER catalyst. However, up to now, studies on the activity of perovskite oxide for HER are scanty, due to the unclear HER mechanism on these materials. More works on perovskites are required for further study of the structure-activity correlation with respect to tunable electronic structures by doping modification, in order to optimize the activity for HER. In spite of the commonly used A-site and/or B-site partial substitution-doping modification, another effective way that is attracting increasing attention is to amend the surface redox chemistry and oxygen deficiency of perovskite oxides via getting A-site cationic deficiencies introduced into their lattice structure. As reported, a significantly improved electrochemical performance has been observed in oxygen reduction reaction with cationic-deficient perovskites (like Ba_1−*x*_Co_0.7_Fe_0.2_Nb_0.1_O_3−*δ*_ (*x* = 0.00–0.15) [15], La_0.6_Sr_0.4−*x*_Co_0.2_Fe_0.8_O_3−*δ*_ (*x* = 0.0–0.2) [16], and PrBa_1−*x*_Co_2_O_5+*δ*_ (*x* = 0–0.08) [16]) as electrocatalysts. However, there has been no reported work yet regarding the catalytic performance of a cation-deficient modification oxide in HER. In this study, the composition of BaCo_0.4_Fe_0.4_Zr_0.1_Y_0.1_O_3−*δ*_ (BaCFZY) was chosen as the parent perovskite oxide for A-site Ba^2+^-deficiency doping because BaCFZY was found to have a high ability for proton uptake by the incorporation of H_2_O (H_2_O + V_O_^∙∙^ + O_O_^x^↔2OH^∙^) and high structural stability in alkaline media, which would be favorable to HER [17]. Meanwhile, as a case study, we evaluated the HER activity of Ba_0.5_Sr_0.5_Co_0.8_Fe_0.2_O_3−*δ*_ (BSCF), one of the most spotlighted perovskite oxides that is assumed to be a strong catalyst candidate for ORR/OER/HER given it is with high catalytic activity [10, 13, 18]. The effects of Ba^2+^ deficiency on the crystal structure, surface chemical properties, microstructure, electrochemical activity, and stability of BaCFZY for HER were carefully investigated. The results suggest that the additional negative charges introduced by an A-site Ba^2+^ deficiency are mainly compensated by the generation of oxygen vacancy. It is helpful to form the low-coordinated active Fe/Co cations which is beneficial to adsorb H_2_O and OH^−^ and to promote the catalytic activity for HER.

## 2. Materials and Methods

### 2.1. Synthesis of BCFZY and BSCF Oxides

Ba_1−*x*_Co_0.4_Fe_0.4_Zr_0.1_Y_0.1_O_3−*δ*_ (Ba_1−*x*_CFZY, *x* = 0–0.05) and BSCF oxides were synthesized by a combined EDTA-citrate complexing sol-gel method. Here, taking BaCFZY as an example, the stoichiometric amounts of Ba (NO_3_)_2_ (AR), Co(NO_3_)_2_·6H_2_O (AR), Fe(NO_3_)_3_·9H_2_O (AR), (Zr(NO_3_)_4_·5H_2_O (AR) and Y(NO_3_)_3_·6H_2_O (AR) were firstly dissolved in deionized water and added into an EDTA-NH_3_·H_2_O solution (pH ≈ 9) under heating and stirring to form an aqueous solution. Then, a certain amount of citric acid-NH_3_·H_2_O solution (pH ≈ 9) was introduced with a molar ratio of 1 : 1 : 2 for EDTA acid : total metal ions : citric acid. The resulting solution was evaporated at 80°C and 150°C in sequence to obtain a dark dry foam-structured precursor. The precursor was made to decompose on a hot plate followed by calcinations in a muffle furnace at 600°C for 5 h and 1050°C for 10 h to yield the desired oxide powders. The commercial catalysts Pt/C were purchased from SangLaiTe.

### 2.2. Material Characterization

Phase structures of as-synthesized Ba_1−*x*_CFZY (*x* = 0.00–0.05) and BSCF powders were characterized by X-ray diffraction measurement (XRD, D/max-2400 Rigaku, Tokyo) with a step size of 0.02° in 2*θ* using the scanning range of 20° to 80° at room temperature. To get more precise details of crystal structures, zero-point correction of XRD patterns was performed based on the X-ray diffraction theory. The morphologies of the catalysts were observed using a field-emission scanning electron microscope (SEM, TESCAN MIRA3). Energy dispersive X-ray spectroscopy (EDX) was carried out to analyze the contained elements in samples. The chemical compositions and surface element states were determined by X-ray photoelectron spectroscopy (XPS) (PHI 5000 VersaProbe II) with Al K*α* as an excitation source.

### 2.3. Electrochemical Measurement

The HER electrochemical activities of the investigated catalyst were evaluated in a three-electrode configuration with the aid of typical thin film rotating disk electrode systems (Pine Instrument Company, USA) on an electrochemical workstation (CHI 760). A standard Hg|HgO (1 M KOH) electrode and a graphite rod electrode were used as the reference and counter electrodes, respectively. The Hg|HgO (1 M KOH) electrode was calibrated with respect to the reversible hydrogen electrode (RHE) according to the Nernst equation (*E* versus RHE = *E* (Hg | HgO) + 0.098 + 0.0592 × pH) in 1 M KOH. The electrolyte was a 1 M KOH aqueous solution (Acros, 99.98%), which was saturated by N_2_ by bubbling N_2_ into it for more than 30 min prior to the test and maintained under a N_2_ atmosphere throughout. The ink of the working electrodes was prepared by the ultrasonic dispersion of 5.0 mg of catalyst, 1.0 mg of acetylene black (AB) carbon, and 33 *μ*L of K^+^-exchanged Nafion solution into the mixture of 500 *μ*L of 2-methoxyl ethanol and 467 *μ*L of tetrahydrofuran for 1 h. Then, 10 *μ*L of the catalyst ink was dropped on the glassy carbon RDE (0.196 cm^2^, area) polished by 50 nm alumina slurry and rinsed by sonicating in pure water. Then, the RDE was rotated at 700 rpm until the film was dry (about 30 min), yielding a catalyst mass loading of 0.255 mg_oxide_cm_disk_^−2^. Before all the measurements, the catalyst was electrochemically activated via cyclic voltammetry in the potential range of −0.9 to −1.65 V (versus Hg|HgO) at 100 mV s^−1^ for 80 cycles with a rotating rate of 1600 rpm. The cyclic voltammetry measurement of HER activity was performed at 10 mV s^−1^ and 1600 rpm. The CV curve was capacity corrected by averaging the forward and reverse currents, and ohmic resistance was corrected according to the following equation: *E*_*iR*−corrected_ = *E* − *iR*, where *i* is the current, and *R* (~0.5 Ω) is the ohmic resistance from an electrolyte measured via electrochemical impedance spectroscopy. The chronopotentiometry was carried out at 20 mA cm_*disk*_^−2^ and 1600 rpm for 2 h. Electrochemical impedance spectroscopy measurement was performed at −0.9 V and − 1.6 V versus Hg|HgO between 100 kHz and 10 Hz with an amplitude of 20 mV.

## 3. Results and Discussion

### 3.1. Phase Structure and Micromorphology

**Figure 1:**
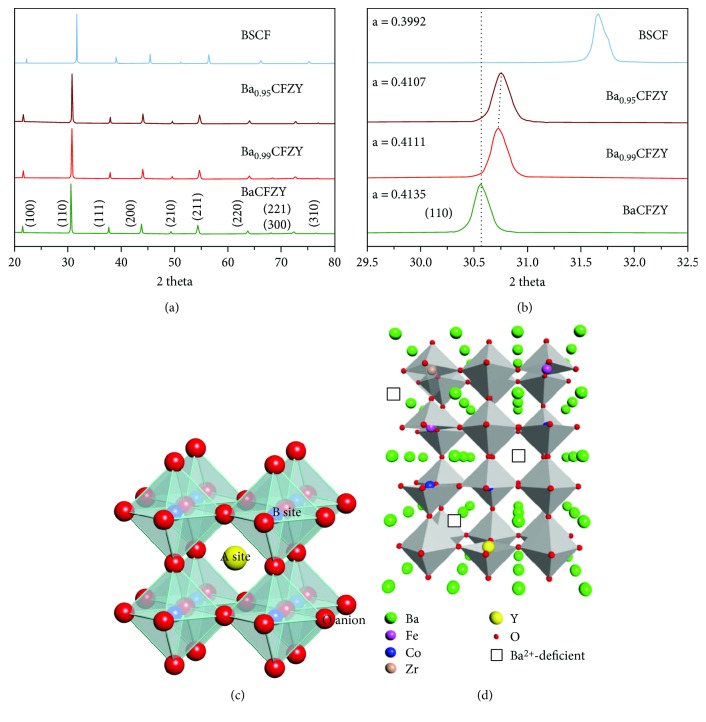
(a) XRD patterns and (b) magnified parts of the as-synthesized BSCF and Ba_1−*x*_CFZY (*x* = 0–0.05) powders calcined at 1050°C for 10 h; (c) crystal structure schematic of a cubic perovskite oxide with the formula of ABO_3_; (d) crystal structure schematic of the Ba^2+^-deficient Ba_1−*x*_CFZY perovskite oxides.

**Figure 2:**
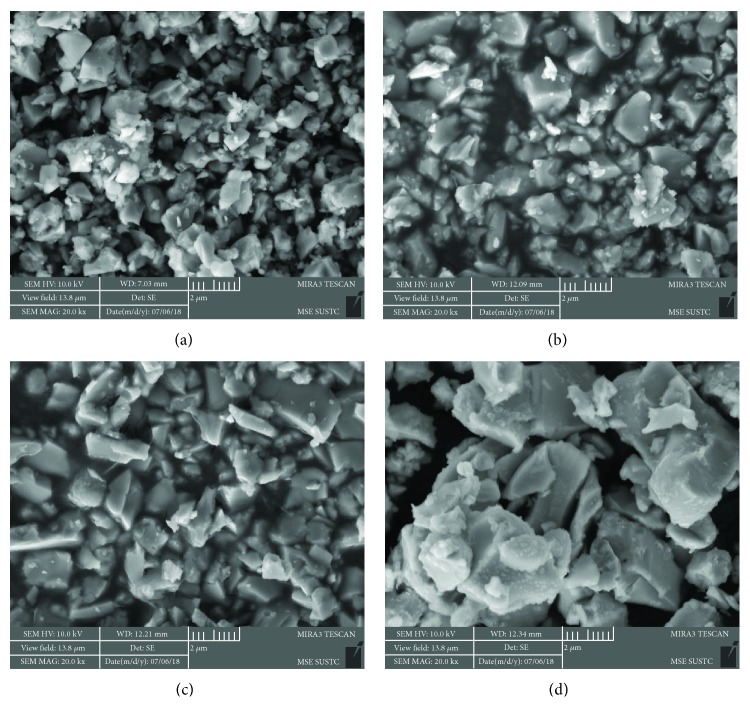
Typical SEM image of as-synthesized (a) BaCFZY, (b) Ba_0.99_CFZY, (c) Ba_0.95_CFZY, and (d) BSCF.

**Figure 3:**
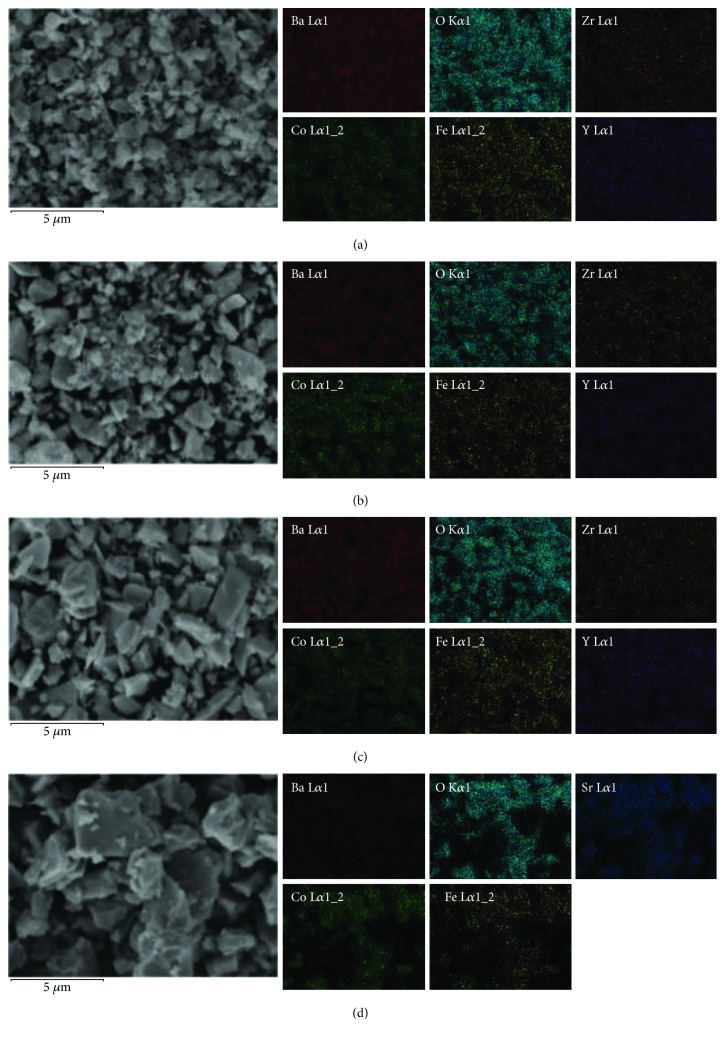
High-resolution SEM image and the corresponding EDX element mapping of (a) BaCFZY, (b) Ba0.99CFZY, (c) Ba0.95CFZY, and (d) BSCF.

Figure 1 shows XRD patterns of BSCF and Ba_1−*x*_CFZY oxides with various Ba^2+^ deficiencies (*x* = 0–0.05) after calcination at 1050°C for 10 h. All the diffraction peaks could be indexed by a cubic *Pm*-3*m* space group without a detectable amount of impurities, indicating that the as-synthesized Ba_1−*x*_CFZY and BSCF powders are single phased and well crystallized. As we know, BSCF is a well-known perovskite oxide with the general formula ABO_3_.  Figure 1(c) illustrates a typical crystal structure schematic of a cubic perovskite oxide. Thereunto, for BSCF Ba^2+^/Sr^2+^ ions are at the A site and Co/Fe ions are situated at the B site (the center of the oxygen octahedron). While in the Ba_1−*x*_CFZY perovskite structure Figure 1(d)), the A site is occupied by 12-fold coordinated Ba^2+^ cations with a larger ionic radius, while the B site is occupied by smaller Co/Fe/Zr/Y ions in 6-fold coordination to the oxygen anions (that is to say Co/Fe/Zr/Y ions are at the center of the oxygen octahedron). Moreover, from the magnified parts of the XRD patterns depicted in Figure 1(b), in comparison with BSCF, the diffraction peaks of Ba_1−*x*_CFZY (*x* = 0–0.05) all shifted to lower 2*θ* angles, indicating an expansion in the perovskite lattice as a result of the introduction of the larger Zr^4+^ (0.72 Å) and Y^3+^ (0.9 Å) ions. In contrast, the diffraction peaks (110) for Ba_1−*x*_CFZY shift slightly to higher angles with the increase of Ba^2+^ deficiency, indicating a lattice shrinkage probably from the increased electrostatic attraction [19]. Besides, the crystal lattice parameter could be obtained by the Bragg diffraction equation and be marked in Figure 1(b). The results indicate that with a higher Ba^2+^ deficiency introduced from *x* = 0 to *x* = 0.05, the lattice constant of Ba_1−*x*_CFZY is decreased slightly from 4.135 Å to 4.107 Å, but it is still larger than that of BSCF (0.399 Å). Figures 2(a) and 3(a) present typical SEM images for Ba_1−*x*_CFZY and BSCF. Unlike the classical perovskite powders of BSCF with an irregularly and aggregate shape [20], BaCFZY presents a uniformly distributed blocky appearance with more edge-like surfaces, which would provide more active sites for HER than that of BSCF. Besides, the corresponding energy-dispersive X-ray elemental (EDX) mapping as shown in Figures 2(c)–2(h) and 3(c)–3(g) suggests the homogeneous distribution of Ba, Co, Fe, Zr, Y, and O for BCFZY as well as Ba, Sr, Co, Fe, and O for BSCF.

### 3.2. X-Ray Photoelectron Spectroscopy Analysis

**Figure 4:**
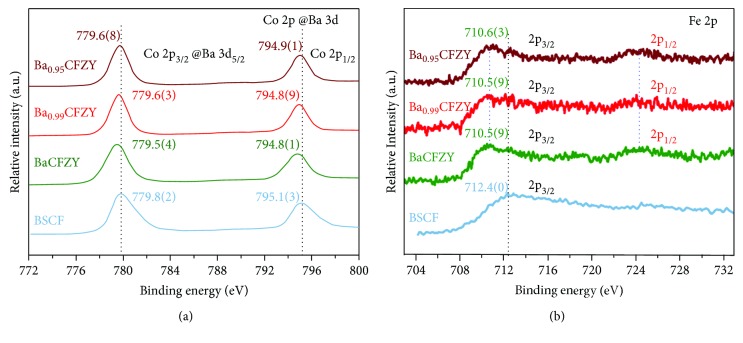
(a) Co 2p and Ba 3d core-level XPS results of BSCF and Ba_1−*x*_CFZY (*x* = 0–0.05). (b) Fe 2p core-level XPS results of BSCF and Ba_1−*x*_CFZY (*x* = 0–0.05).

To analyze the influence of the surface properties of BSCF and Ba_1−*x*_CFZY (*x* = 0–0.05) on HER, the chemical states of the active elements of Co and Fe were measured by XPS and normalized with a C 1s peak to 284.6 eV. Via the narrow spectra of high-resolution XPS as shown in Figure 4, the peaks of Fe 2p_3/2_, Fe 2p_1/2_, and Co 2p_1/2_ can be clearly identified in all samples, while it is difficult to separate out the Co 2p_2/3_ peaks due to the overlap between Co 2p_3/2_ and Ba 3d_5/2_ main lines [14]. Meanwhile, the negative shift of the characteristic peaks of Fe 2p_3/2_, Fe 2p_1/2_, and Co 2p_1/2_ together with Co 2p_3/2_ @Ba3d_2/5_ can be observed, indicating that the reduction of both Co and Fe cations in Ba_1−*x*_CFZY as compared to BSCF [13, 21]. Additionally, both of the Co 2p and Fe 2p peak positions of Ba_1−*x*_CFZY (*x* = 0–0.05) are almost invariant, and only a slight increment shift (~0.1 eV for Co and ~0.06 eV for Fe) from BaCFZY to Ba_0.95_CFZY was detected. The little change of peak position implies that the additional negative charges introduced by the A-site Ba^2+^ deficiency are barely compensated by the oxidation of B-site Co/Fe ions to a higher valence state in Ba_1−*x*_CFZY (*x* = 0–0.05), but mainly by the generation of oxygen vacancy. Namely, the introduction of Ba^2+^ deficiency will create more oxygen vacancy in the lattice (like the case of (Ba_0.5_Sr_0.5_)_1−*x*_Co_0.8_Fe_0.2_O_3−*δ*_ [22], PrBa_1−*x*_Fe_2_O_5+*δ*_ [23], and La_0.6_Sr_0.4−*x*_Co_0.2_Fe_0.8_O_3−*δ*_ [16]) which is helpful to form the low-coordinated activity of Fe/Co cations which facilitates the adsorption of H_2_O and OH^−^, and is expected to be beneficial to promote the catalytic activity for HER.

### 3.3. Catalytic Activity for HER

**Figure 5:**
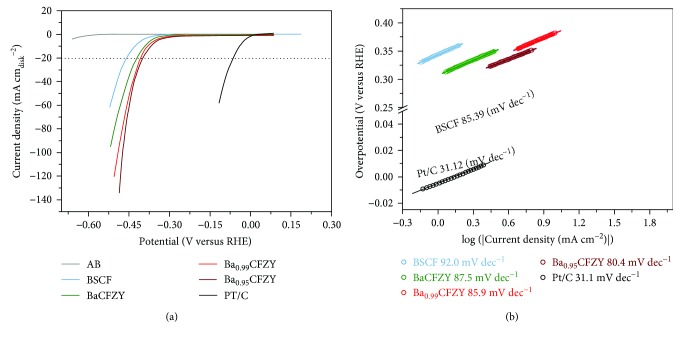
(a) Polarization curves and (b) the corresponding Tafel plots of BSCF, Ba_1−*x*_CFZY (*x* = 0–0.05), and commercial Pt/C catalysts. The background HER activity of a conductive acetylene black- (AB-) supported GC electrode is shown for reference.

To evaluate the electrochemical catalytic activity of Ba_1−*x*_CFZY (*x* = 0–0.05) for HER, the catalytic performance of BSCF, commercial Pt/C, and acetylene black (AB) was measured simultaneously for comparison. All of the polarization curves described in Figure 5(a) were capacity corrected by averaging the forward and backward currents of the CV curves, and were normalized to the geometric area of the GC electrode. It can be seen that Pt/C exhibits superior HER activity in alkaline media with a near-zero onset overpotential, and the conductive acetylene black shows negligible HER activity in the investigated potential range. All Ba_1−*x*_CFZY samples show much smaller onset potential than that of BSCF. (e.g., to achieve a current density of 0.5 mA cm^−2^ and 1 mA cm^−2^, the overpotential needed is 27 mV and 254 mV for Ba_0.95_CFZY, while for BSCF the overpotential is 270 mV and 306 mV, resp.). The HER performance of Ba_1−*x*_CFZY was found to be enhanced by A-site Ba^2+^-deficiency doping. The activity of perovskite oxides Ba_1−*x*_CFZY in catalyzing HER was further evaluated using the overpotential required to deliver an electrode current density of 10 mA cm^−2^ (*η*_10_)—a desirable current density on the basis of 10% solar-to-fuel conversion efficiency. Ba_0.95_CFZY shows the lowest *η*_10_ of 360 mV in Ba_1−*x*_CFZY, which is much lower than that of BSCF (430 mV). Although this value is larger than that of commercial Pt/C, it is comparable to many other homogenous-structured non-Pt catalysts (noble metal-free or transition-metal complex-based catalysts) for HER in alkaline medium, such as Ni [24], Co-NRCNTs [6], and Mn_1_Ni_1_ [25]. Moreover, the Tafel plots depicted in Figure 5(b) were calculated to get an insight into the HER kinetic processes of the catalysts. The Pt/C catalyst shows a Tafel slope of 31.2 mV dec^−1^, which is consistent with the reported values [11, 26]. The Tafel slope of Ba_0.95_CFZY is 80.4 mV dec^−1^, which is lower than those of Ba_0.99_CFZY (85.9 mV dec^−1^), BaCFZY (87.5 mV dec^−1^), and BSCF (92.0 mV dec^−1^). The substantially enhanced HER activity achieved by the introduction of Ba^2+^ deficiency into the A site is further confirmed by the gradually decreased Tafel slope, demonstrating that the Ba^2+^-deficiency doping plays a positive role in facilitating the kinetics process of water splitting. Additionally, the rate-limited step of HER can be assessed from the Tafel slope (slopes of ≈120, 40, and 30 mV dec^−1^ corresponding to the Volmer, Heyrovsky, and Tafel reaction limitations, respectively [5, 9, 27]). It can be seen that all the observed Tafel slopes for BSCF and Ba_1−*x*_CFZY catalysts are between 40 and 120 mV dec^−1^, suggesting that electrochemical desorption is the rate-limited step in the catalysts and the HER process possibly occurs via a Volmer-Heyrovsky mechanism.

**Figure 6:**
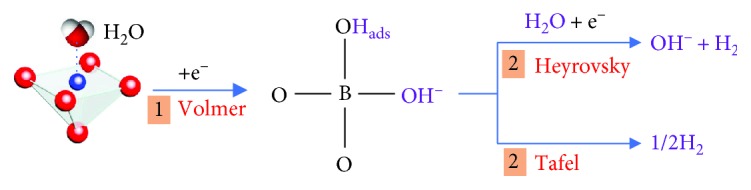
Schematic diagram of the HER reaction pathway on perovskite oxide.

As well known, electrocatalytic HER proceeds through two charge-transfer steps in alkaline media. The first step is a Volmer reaction (H_2_O + e^−^ → H_ads_ + OH^−^), in which the water molecule adsorbed on the catalyst surface is ionized and transferred to be H_ads_. The second step is either a Heyrovsky reaction (H_2_O + H_ads_ + e^−^ → H_2_ + OH^−^) or a Tafel reaction (H_ads_ + H_ads_ → H_2_) [26]. Both of the HER pathways (Volmer-Heyrovsky or Volmer-Tafel pathway) involved the adsorption of water molecules on the active sites, electrochemical reduction of adsorbed water molecules into adsorbed hydroxyl ions (OH^−^) and H_ads_, desorption of OH^−^ to refresh the catalyst surface, and formation of H_ads_ for H_2_ generation [7]. Therefore, according to the HER mechanism, the highly efficient HER process means that the catalyst has good H_2_O adsorption abilities, strong affinity to H_ads_, and facile desorption of OH^−^ on the active site. More importantly, early fundamental studies suggested that, in alkaline medium, OH^−^ competes with H_ads_ for surface active sites of metal-based catalysts, seriously poisoning the electrode and reducing overall rates [6]. Thus, the water-splitting process may also be influenced by the OH_ads_ species on the electrode [12, 28]. It is reported that the low-coordinated active atoms on the surface can serve as active sites accessible to the OH_ads_ species [28, 29]. Consequently, the increase of active atoms adjacent to oxygen vacancies will be beneficial for OH^−^ adsorption (generated from H_2_O splitting) and can leave alone a nearby anion ion (O^2−^) site for H_ads_ (Figure 6). These low-coordinated active atoms and adjacent oxygen ions may play the role of heterojunctions that synergistically facilitate the Volmer process and thus render stimulated HER catalytic activity.

**Figure 7:**
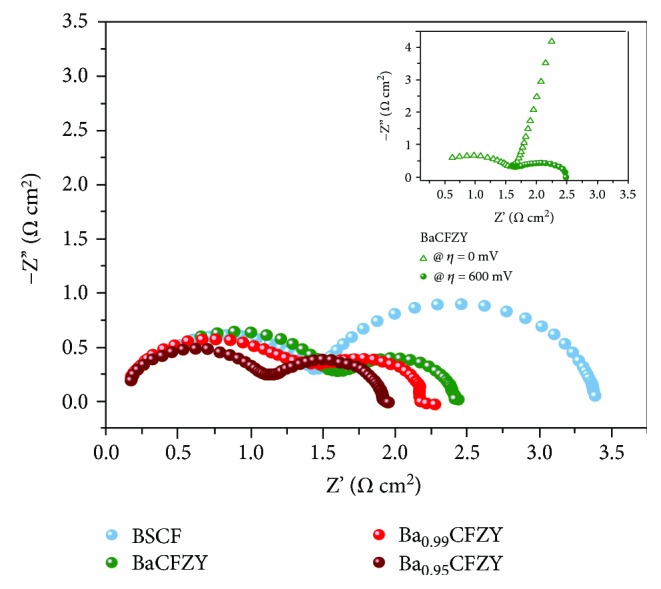
EIS Nyquist plots of BSCF and Ba_1−*x*_CFZY (*x* = 0–0.05) catalysts collected at HER overpotential of 600 mV. The inset in Figure 7 shows the EIS Nyquist plots of the BaCFZY catalyst at various overpotentials.

As we can conclude from the XPS results in Figure 4, the enhanced activity of Ba_0.99_CFZY could be assigned to the formation of oxygen vacancies around active ions Co/Fe. Moreover, as the charge transfer resistance (*R*_*ct*_) has a direct bearing on HER processes, electrochemical impedance spectroscopy was conducted at an overpotential of 600 mV to investigate the electrode kinetics of HER process for BSCF and Ba_1−*x*_CFZY catalysts. As can be observed from Nyquist plots in Figure 7, the introduction of Ba^2+^ deficiency results in an obvious semicircle decrease in the low-frequency zone, signifying the smaller *R*_*ct*_ of Ba_1−*x*_CFZY as a result of Ba^2+^-deficiency doping. The truth that all *R*_*ct*_ of Ba_1−*x*_CFZY are smaller than that of BSCF, indicates a faster electron transferring and more facile HER kinetics at the electrode/electrolyte interface of Ba_1−*x*_CFZY [13]. Thus, the facile method of introducing A-site deficiency in perovskite lattice could be an effective way of promoting HER catalytic activity.

**Figure 8:**
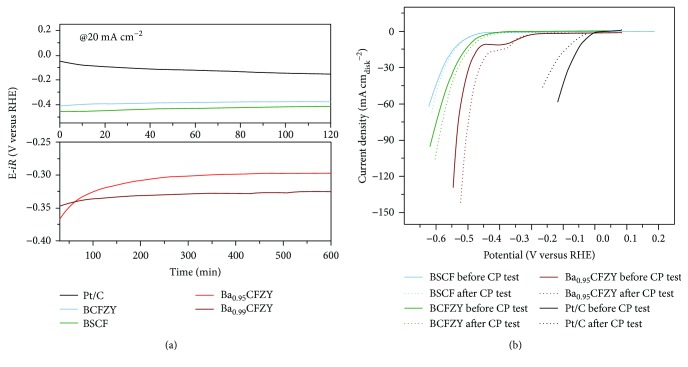
(a) Chronopotentiometry test curves of BSCF, Ba_1−*x*_CFZY, and commercial Pt/C catalysts at a constant cathodic current density of 20 mA cm^−2^. (b) Comparison of the HER activity curves of BSCF, Ba_1−*x*_CFZY, and commercial Pt/C catalysts before and after chronopotentiometry measurement.

Stability is another important factor in the development of advanced electrocatalysts. To assess this, a chronopotentiometric (CP) test (Figure 8) was carried out and the voltammograms (Figure 8) before and after the CP test for 2 h at 20 mA cm^−2^ were compared. Pt/C exhibits the highest initial activity but undergoes a rapid degradation during the CP test, corresponding to its poor HER durability as reported [14]. In sharp contrast, BaCFZY even exhibits a decreasing operating overpotential over the same testing period, demonstrating its superior stability in the long-term electrochemical process. Meanwhile, the current density of the voltammogram of BaCFZY even increases slightly after 2 h of catalyzing HER, further indicating the stable HER electrocatalysis of BaCFZY in basic solutions. These results confirm the potentials of the Ba_1−*x*_CFZY perovskite oxide as an efficient, stable, and economic HER catalyst.

In conclusion, A-site Ba^2+^-deficiency doped BaCo_0.4_Fe_0.4_Zr_0.1_Y_0.1_O_3−*δ*_ oxides, Ba_1−*x*_Co_0.4_Fe_0.4_Zr_0.1_Y_0.1_O_3−*δ*_ (Ba_1−*x*_CFZY, *x* = 0.00–0.05) have been synthesized and evaluated as a kind of new electrocatalysts for HER in alkaline solutions. As a case study, Ba_0.5_Sr_0.5_Co_0.8_Fe_0.2_O_3−*δ*_ (BSCF) oxides have also been synthesized and studied. The as-synthesized Ba_1−*x*_CFZY and BSCF oxides are well-crystallized single phases, indexed with the cubic space group of *Pm*-3*m*. All Ba_1−*x*_CFZY samples show a much lower onset potential and overpotential and smaller Tafel slope than that of BSCF. The HER performance of BaCFZY can be enhanced by simple A-site Ba^2+^-deficiency doping. Electrochemical desorption is the rate-limited step on Ba_1−*x*_CFZY catalyst surfaces and the HER process may occur via a Volmer-Heyrovsky mechanism. Furthermore, Ba_1−*x*_CFZY exhibits superior stability in the long-term of the electrochemical process compared with commercial Pt/C. The enhanced HER performance may originate from the increased oxygen vacancies around active Co/Fe ions on the surface of Ba_1−*x*_Co_0.4_Fe_0.4_Zr_0.1_Y_0.1_O_3−*δ*_ induced by Ba^2+^-deficiency doping into the A site. These low-coordinated active atoms and contiguous oxygen ions may play the role of heterojunctions that synergistically facilitate the Volmer process and thus render stimulated HER catalytic activity, corresponding to a faster electron transfer and more facile HER kinetics at the electrode/electrolyte interface. The method of introducing A-site deficiency in perovskite lattice could be a facile and effective way to promote HER catalytic activity and this work sheds light on perovskite oxides as electrocatalysts for HER applications.

## Data Availability

The data used to support the findings of this study are available from the corresponding author upon request.
